# An adenoviral vector encoding an inflammation-inducible antagonist, HMGB1 Box A, as a novel therapeutic approach to inflammatory diseases

**DOI:** 10.1128/mbio.03387-24

**Published:** 2024-12-19

**Authors:** Kari Ann Shirey, John Joseph, Lynda Coughlan, Haye Nijhuis, Alan W. Varley, Jorge C. G. Blanco, Stefanie N. Vogel

**Affiliations:** 1 Department of Microbiology and Immunology, University of Maryland, School of Medicine, Baltimore, Maryland, USA; 2 Sigmovir Biosystems Inc., Rockville, Maryland, USA; 3 Center for Vaccine Development and Global Health (CVD), University of Maryland, School of Medicine, Baltimore, Maryland, USA; 4 Department of Medical Microbiology and Infection Prevention, Amsterdam University Medical Center, Location AMC, University of Amsterdam, Amsterdam Institute for Immunology and Infectious Diseases, Amsterdam, the Netherlands; 5 Independent Researcher, Plano, Texas, USA; University of Pennsylvania, Philadelphia, Pennsylvania, USA

**Keywords:** TLR4, MD-2, HMGB1, Box A, influenza, LPS, mice, cotton rats, adenovirus

## Abstract

Influenza, as well as other respiratory viruses, can trigger local and systemic inflammation resulting in an overall “cytokine storm” that produces serious outcomes such as acute lung injury (ALI) or acute respiratory distress syndrome (ARDS). We hypothesized that gene therapy platforms could be useful in these cases if the production of an anti-inflammatory protein reflects the intensity and duration of the inflammatory condition. The recombinant protein would be produced and released only in the presence of the inciting stimulus, avoiding immunosuppression or other unwanted side effects that may occur when treating infectious diseases with anti-inflammatory drugs. To test this hypothesis, we developed AdV.C3-Tat/HIV-Box A, an inflammation-inducible cassette that remains innocuous in the absence of inflammation but releases HMGB1 Box A, an antagonist of high mobility group box 1 (HMGB1), in response to inflammatory stimuli such as lipopolysaccharide (LPS) or influenza virus infection. We report here that this novel inflammation-inducible HMGB1 Box A construct in a non-replicative adenovirus (AdV) vector mitigates lung and systemic inflammation therapeutically in response to influenza infection. We anticipate that this strategy will apply to the treatment of multiple diseases in which HMGB1-mediated signaling is a central driver of inflammation.

**IMPORTANCE:**

Many inflammatory diseases are mediated by the action of a host-derived protein, HMGB1, on Toll-like receptor 4 (TLR4) to elicit an inflammatory response. We have engineered a non-replicative AdV vector that produces HMGB1 Box A, an antagonist of HMGB1-induced inflammation, under the control of an endogenous complement component C3 (C3) promoter sequence, that is inducible by LPS and influenza *in vitro* and *ex vivo* in macrophages (Mϕ) and protects mice and cotton rats therapeutically against infection with mouse-adapted and human non-adapted influenza strains, respectively, *in vivo*. We anticipate that this novel strategy will apply to the treatment of multiple infectious and non-infectious diseases in which HMGB1-mediated TLR4 signaling is a central driver of inflammation.

## INTRODUCTION

Influenza virus infection causes serious disease worldwide and can lead to a massive death toll during pandemics, particularly, when followed by secondary bacterial infection (1–3). Prediction of which influenza strains to incorporate in each year’s vaccine, including unexpected strain dominance, has led to efforts to develop a “universal vaccine” (4–7). Antiviral drugs are limited by their need to be administered early after infection (8) and the appearance of drug-resistant strains (8, 9), although at a low frequency. Although the disease is initiated by influenza replication resulting in airway epithelial damage, the severe inflammatory response that follows metabolic stress in innate immune cells (e.g., Mϕ) elicits a “cytokine storm” that leads to acute lung injury (ALI) or the more severe acute respiratory distress syndrome (ARDS) (10, 11), also seen in SARS-CoV-2 (12).

Our initial finding that TLR4^−/−^ mice are highly refractory to lethal influenza infection (13, 14) led to the hypothesis that TLR4 antagonist therapy would mitigate disease by blunting the “cytokine storm.” Eritoran (Eisai, Inc.), a potent lipid A analog antagonist that acts by competitive inhibition of the TLR4 co-receptor, MD-2 (14), failed in Phase 3 clinical trials for all-cause sepsis (15). However, we reasoned that influenza, which targets the lung and leads to a “cytokine storm,” might be more amenable to therapeutic TLR4 antagonism. Eritoran treatment of mice infected with an LD_90_ of mouse-adapted influenza strain A/PR/8/34 (PR8) starting 2 days post-infection (p.i.) for 5 days, blunted inflammatory cytokines and lung histopathology, with significant survival, even when treatment was delayed until 6 days p.i. (14, 16–19). These findings were confirmed in cotton rats (CR), *Sigmodon hispidus*, that, in contrast to mice, are susceptible to non-adapted human respiratory viruses (20–22). Eritoran treatment of mice or CR also blunted the enhanced disease observed when influenza is followed by secondary Gram-positive infection (23). We have since shown that many TLR4 antagonists that are structurally unrelated to Eritoran and act by distinct mechanisms are highly effective therapeutically in influenza-infected mice and CR (24). However, Eritoran synthesis is very complex (25, 26), and continuous or repeated i.v. dosing is required in murine or human endotoxicity, sepsis, and influenza infection (14, 26, 27).

Importantly, influenza does not express “pathogen-associated molecular patterns” (PAMPs) that activate TLR4. We found that influenza-induced lung inflammation and lethality are mediated by host-derived HMGB1 (17). HMGB1, a nuclear protein released from dying cells (28), was first identified as a key mediator of endotoxicity and sepsis (29–31). Like the prototype TLR4 PAMP, LPS, the disulfide-HMGB1 isoform is a “danger-associated molecular pattern (DAMP)” that activates TLR4 signaling by binding the TLR4 co-receptor, MD-2 (32–36).

However, other mechanisms of TLR4-dependent HMGB1 signaling have been proposed *via* CD14 (37) or the HMGB1 receptor, receptor for advanced glycation end products (RAGE) (38). In contrast to TLR4^−/−^ mice (13, 14, 39), both CD14^−/−^ (14) and RAGE^−/−^ mice succumb to PR8, although RAGE^−/−^ mice exhibit an extended mean time to death (40; Fig. S1). Therefore, the direct HMGB1-MD-2 binding mechanism seemed most likely. To test this hypothesis, the treatment of mice therapeutically with P5779, a small molecule inhibitor that competitively inhibits HMGB1 binding to MD-2 and blocks TLR4 signaling (32, 34), protected PR8-infected mice comparably to Eritoran (17). However, the high concentrations of P5779 required for protection are not clinically achievable. In CR infected with human influenza strains, disease severity based on lung pathology and cytokine production correlated with serum HMGB1 levels, and were reduced by Eritoran treatment (23, 41). These data, and reports that HMGB1 underlies many other inflammatory diseases (42–49), suggest that HMGB1-mediated TLR4 signaling is a common denominator.

HMGB1 contains two ~80 amino acid domains, Box A and Box B (50). Box A binds TLR4 and stabilizes the interaction of Box B with MD-2 that triggers TLR4-dependent cytokine-stimulating activity of intact HMGB1 (34). Recombinant HMGB1 Box A (rBox A) competitively inhibits disulfide-HMGB1-induced activation of TLR4/MD-2 signaling by binding with high affinity to TLR4, thereby displacing native HMGB1 (34). *In vitro,* bacterially derived rBox A inhibited cytokine release from HMGB1-treated murine Mϕ (30). rBox A also reduced inflammation associated with allograft rejection (51), ischemia (52, 53), sterile injury (34), and others. However, in a model of intratracheal (i.t.) LPS, even 600 µg/mouse of rBox A was only partially protective (54), and this same dose administered multiple times to mice that received LPS systemically or during cecal ligation and puncture, induced only partial protection (30). Our data showing a lag of 2–4 days for induction of circulating HMGB1 in response to influenza (41), coupled with the partial efficacy of Eritoran as late as 6 days p.i. (14), suggests there is a therapeutic window in influenza-induced ALI.

Herein, we report the development of a novel, inflammation-inducible rBox A expression cassette encoded by a non-replicative AdV vector, “AdV.C3-Tat/HIV-Box A,” that mitigates lung and systemic inflammation when administered therapeutically to influenza-infected animals. We anticipate that this approach may be tested using diverse viral vectors, and will apply to the treatment of multiple diseases in which HMGB1-mediated TLR4/MD-2 signaling is a central driver of inflammation (45).

## RESULTS

### Engineering AdV.C3-Tat.HIV-Box A vectors

**Fig 1:**
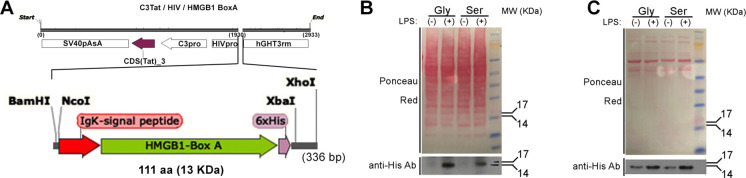
(A) Structure of AdV.C3-Tat/HIV-Box A cloned under control of a two-component, inflammation-driven, AdV expression system (C3-Tat/HIV, top), our chimeric DNA construct encodes the HMGB1 Box A protein (green arrow) flanked by a secretory peptide (N-terminus, red arrow) and a C-terminal, 6x-His tag (purple arrow) (total 111 aa). (B) Detection of HMGB1 Box A produced in cell lysates of CR peritoneal Mϕ infected *in vitro* with AdV.C3-Tat/HIV-Box A^Gly^ or -Box A^Ser^ (MOI = 1) and then treated with LPS (10 ng/mL, 18 h). (C) Detection of secreted HMGB1 Box A^Gly^ or HMGB1 Box A^Ser^ by CR BAL Mϕ obtained 24 h after i.n. infection with 10^7^ PFU of the indicated AdV.C3-Tat/HIV-Box A vector. BAL Mϕ were harvested 24 h after infection with AdV vectors, then cultured and treated with LPS for 24 h, followed by the analysis of culture supernatants by WB.

Figure 1A illustrates that the AdV vector expresses the protein of interest (rBox A) under the control of a two-component expression system developed by Varley and colleagues (55–57). The construct contains an inflammation-activated promoter region (mouse C3 gene promoter) that, in response to inflammatory insults, drives the production of HIV transactivator of transcription (*Tat*), that induces transcription of the gene of interest through its HIV LTR promoter, inserted into a non-replicating AdV vector. This inflammation-inducible cassette was first used successfully to express luciferase (Luc) *in vivo* (55–57) in response to i.p. LPS or turpentine, and later, to mitigate joint inflammation in rodent models of arthritis by intraarticular expression of IL-1R antagonist (58) or IL-10 (59). We hypothesized that in mice and CR challenged with LPS or infected with influenza, treatment with our engineered vectors, “AdV.C3-Tat/HIV-Box A^Ser^” or “AdV.C3-Tat/HIV-Box A^Gly^,” would produce rBox A to mitigate ALI by antagonizing TLR4-mediated inflammation induced by HMGB1, while “AdV.C3-Tat/HIV-Luc” would secrete luciferase and serve as a negative control.

Several modifications to the Box A sequence were made before insertion into the original AdV construct based on predictions that would facilitate secretion, purification, and detection: (i) The N-terminus of the Box A sequence was placed in tandem with the IgK signal peptide sequence to enhance secretion because, due to the lack of a leader sequence, Box A cannot be actively secreted through the endoplasmic reticulum (ER)-Golgi secretory pathway (60, 61); (ii) The predicted glycosylation site within the wild-type (WT) sequence, “NFS” (resulting in rBoxA^Ser^), was mutated to “NFG” (resulting in non-glycosylatable rBox A^Gly^), to prevent retention of rBox A protein in the ER; and, (iii) A 6X-His tag sequence was added to the Box A C-terminus to facilitate detection by western blot (WB) and permit rBox A purification by Ni^++^ columns.

Comparable protein expression of rBox A^Ser^ and rBox A^Gly^ constructs was observed in transient transfection experiments using Expi293F cells (data not shown). Optimized Box A expression constructs were transferred from a pENTR 1A dual selection vector to the final AdV vector by *in vitro* homologous recombination with a plasmid containing an E1/E3 deleted (non-replicating) AdV backbone using Gateway cloning. Genome sequences were confirmed, and viruses were rescued following Pacl genome linearization and transfection into TRex293 cells. AdV recombinants encoding Box A^Ser^, Box A^Gly^, and luciferase (Luc) were amplified, purified by two rounds of CsCl ultracentrifugation, titrated for infectious titers (all virus stocks had a viral titer of >10^11^ PFU/mL) and physical titers (>10^12^ vp/mL)) (62, 63).

### Inducibility of the non-replicating AdV.C3-Tat/HIV-Box A promoter system by inflammatory stimuli

CR peritoneal Mϕ were infected with AdV.C3-Tat/HIV-Box A^Gly^ or AdV.C3-Tat/HIV-Box A^Ser^ at a multiplicity of infection (MOI) of 1. After 24 h, Mϕ were treated with medium or LPS (10 ng/mL) for 18 h. Proteins were separated on a 4%–12% SDS-PAGE and transferred to a membrane that was stained with Ponceau red for total protein (Fig. 1B, top) and WB with anti-His antibody (Fig. 1B, bottom). AdV-infected CR Mϕ expressed both WT (Ser) and mutated (Gly) Box A proteins only when stimulated with LPS (with the Box A^Gly^ showing slightly higher expression) at the predicted 13 kDa MW by WB. CR bronchoalveolar lavage (BAL) Mϕ obtained 24 h after i.n. inoculation with AdV.C3-Tat/HIV-Box A^Gly^ or AdV.C3-Tat/HIV-Box A^Ser^ (10^7^ PFU/CR), were stimulated *ex vivo* with LPS (Fig. 1C). While low levels of Box A-His proteins were detected in culture supernatants of BAL Mϕ stimulated with medium only, levels of both Box A variants increased comparably upon LPS stimulation (Fig. 1C), indicating that glycosylation of rBox A does not impede secretion. Low levels of rBox A in supernatants of the medium-treated Mϕ are likely attributable to low-level inflammation *in vivo* that stimulates the vectors’ C3 promoter to induce the His-tagged protein.

**Fig 2:**
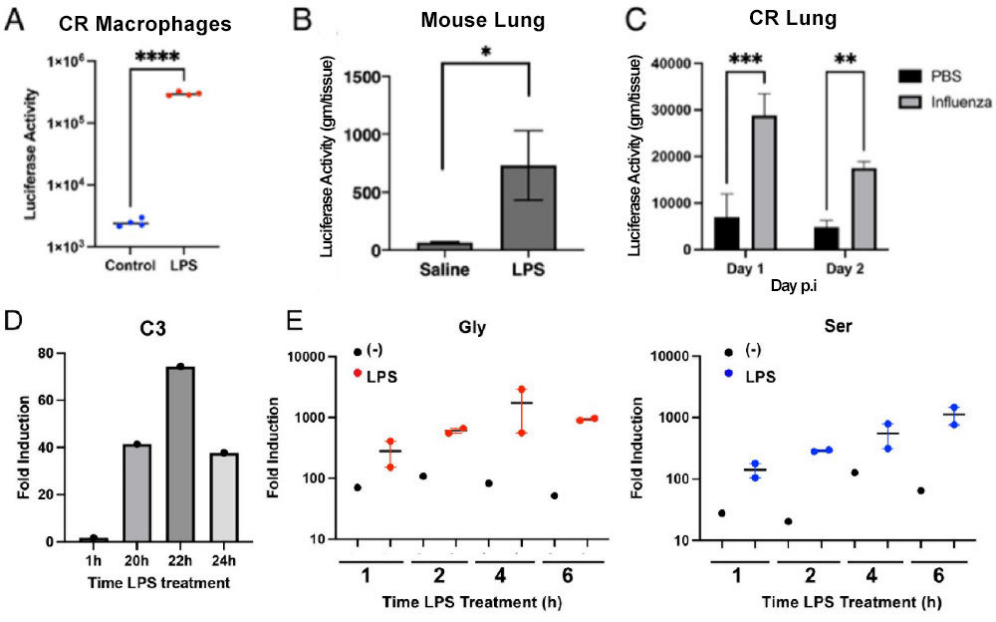
(A) CR peritoneal Mϕ were treated with AdV.C3-Tat/HIV-Luc for 24 h and half the samples were exposed to LPS (10 ng/mL) for an additional 24 h. Luciferase activity was measured in cell lysates (*n* = 4, Student’s *t* test, ****P < 0.0001). (B) Mice were treated with AdV.C3-Tat/HIV-Luc i.n. and challenged with saline or LPS i.t. (10 µg/mouse); mice were sacrificed at 18 h post-challenge, and lung luciferase levels were measured; *P* < 0.05, Student’s *t* test. (C) CR were treated i.n. with AdV.C3-Tat/HIV-Luc (10^5^ PFU/CR) and challenged i.n. with PBS or influenza A(H3N2) virus (10^7^ TCID_50_/animal). CR were sacrificed at the indicated times p.i. with A(H3N2) (10^5^ TCID_50_/CR) and luciferase expression measured in lung homogenates. (*n* = 3–5/group; ANOVA, ***P < 0.0001; **P < 0.01). (D) CR peritoneal Mϕ were exposed to LPS (10 ng/mL) for the indicated times. Expression of the endogenous cotton rat C3 gene was determined by quantitative real-time (qRT)-PCR. (E) CR peritoneal Mϕ were transduced with AdV.C3-Tat/HIV-Box A^Gly^ or AdV.C3-Tat/HIV-Box A^Ser^ using an MOI = 1. Twenty-four hours post-transduction, cells were exposed to medium only (black symbols), LPS (Gly—red symbols; Ser—blue symbols) for the indicated time periods. Expression of the vector-based Box A mRNA expression was measured by qRT-PCR to show the kinetics of Adv.C3-Tat/HIV/Box A expression of the C3 mRNA post-LPS treatment.

**Fig 3:**
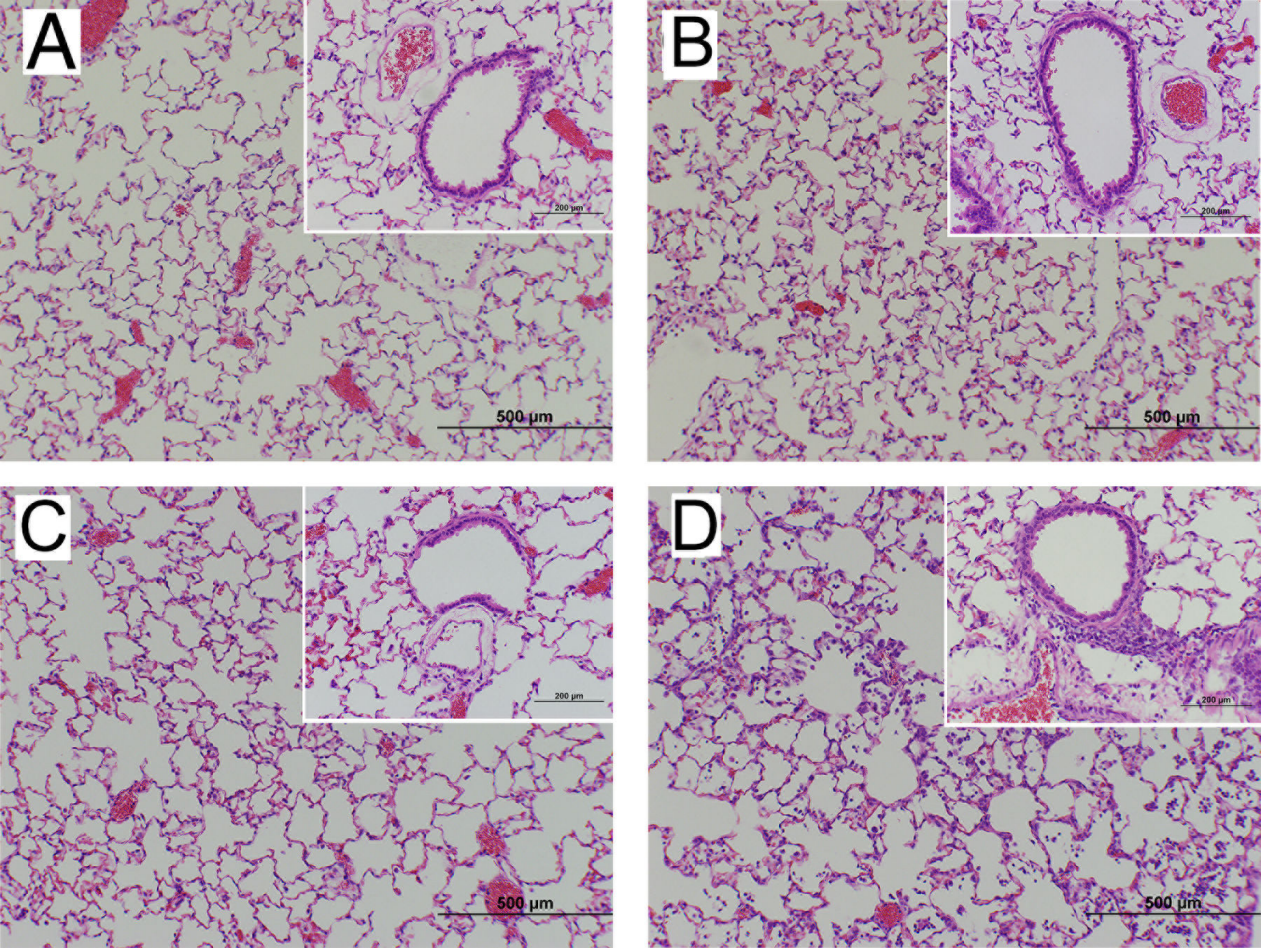
CR were treated i.n. with PBS (A; 100 µL), or with AdV.C3-Tat/HIV-Luc (B and C; 10^7^ PFU/CR in 100 µL) and sacrificed on days 1 (**A, B**) or day 3 p.i. (**C**), showing undetectable lung inflammation in CR treated with AdV.C3-Tat/HIV-Luc in contrast to CR infected with A(H3N2) (10^7^ TCID_50_/CR) and harvested on day 3 p.i. (**D**). Magnification, 100×. Insets show details of bronchi, 200×.

To confirm the inflammation-inducible activity of the promoter *in vitro*, CR Mϕ were infected with AdV.C3-Tat/HIV-Luc (MOI = 1) 24 h prior to LPS stimulation for an additional 24 h, resulting in strong luciferase induction (Fig. 2A). *In vivo*, mice were administered AdV.C3-Tat/HIV-Luc i.n. (10^5^ PFU/mouse) 3 days prior to the LPS challenge (10 µg/mouse i.t.). After 18 h, a time when LPS induces significant lung pathology and cellular infiltration (39), luciferase levels in lung homogenates were significantly increased only in response to LPS (Fig. 2B), indicating inflammation-induced activation of the construct *in vivo*. Next, CR were treated i.n. with AdV.C3-Tat/HIV-Luc (10^5^ PFU/CR) and infected 3 days later with human influenza A(H3N2) (A/Wuhan/359/95; 10^7^ TCID_50_/CR). Lung luciferase activity was significantly increased 1 and 2 days p.i. (Fig. 2C). These data correlated with endogenous C3 gene expression in CR Mϕ treated with LPS (Fig. 2D) and the early kinetics of expression of Box A^Gly^ and Box A^Ser^ mRNA in in CR Mϕ transduced with AdV-C3.Tat/HIV-Box A^Gly^ or AdV-C3.Tat/HIV-Box A^Ser^ followed by LPS treatment (Fig. 2E). Importantly, (i) mice/CR treated with AdV.C3-Tat/HIV-Luc alone (without inflammatory induction) showed low luciferase expression (Fig. 2B and C) and (ii) treatment of CR i.n. with either PBS (Fig. 3A) or with 10^7^ PFU of AdV.C3-Tat/HIV-Luc for 1 day (Fig. 3B) or 3 days (Fig. 3C) failed to induce lung inflammation, in contrast to the strong alveolitis and interstitial pneumonia seen 3 days p.i. with A(H3N2) (Fig. 3D). Thus, our AdV.C3-Tat/HIV vectors are inducible by ALI-inducing challenges and safe *in vivo* in the absence of an inflammatory stimulus.

### AdV.C3-Tat/HIV-Box A therapy protects mice against lethal influenza

**Fig 4:**
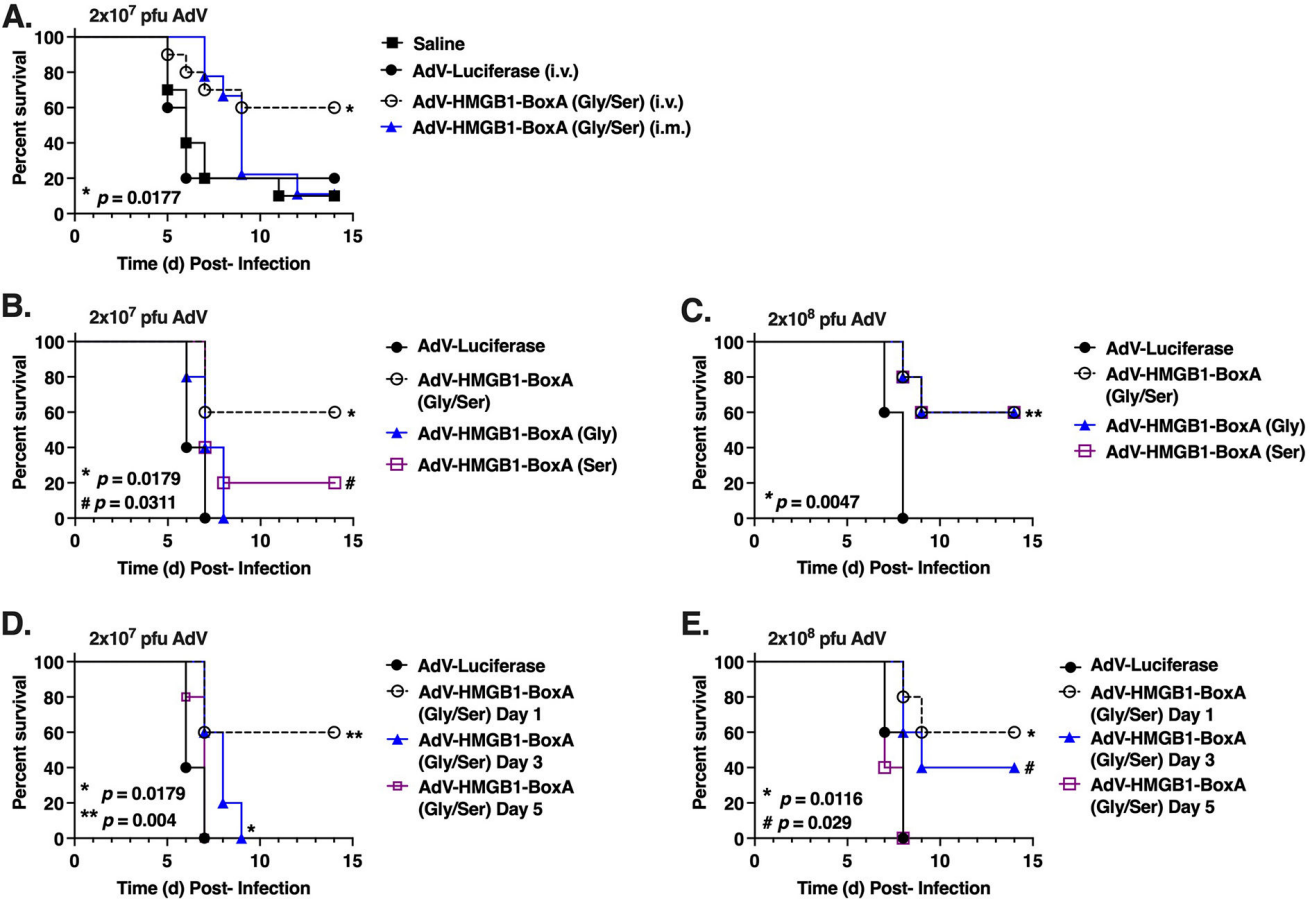
(A) C57BL/6J mice were infected on day 0 with PR8 (LD_90_). Twenty-four hours later, mice were treated with saline (i.m.), AdV.C3-Tat/HIV-Luc (i.v.), or an equal mixture of AdV.C3-Tat/HIV-Box A variants (2 × 10^7^ PFU/mouse) administered either i.m or i.v. Data are from two separate experiments with 5 mice/treatment/experiment. Results from AdV-Luc-treated mice were obtained from 5 mice in a single experiment. (B) Mice were infected as in panel A. Twenty-four hours later, mice were treated i.v. with AdV.C3-Tat/HIV-Luc (2 × 10^7^ PFU/mouse), an equal mixture of AdV.C3-Tat/HIV-Box A variants, or the individual AdV.C3-Tat/HIV-Box A variants (Gly or Ser) (2 × 10^7^ PFU/mouse). Data are from two separate experiments with 5 mice/treatment/experiment. (C). Mice were infected as in panel A. Mice were treated i.v. with AdV.C3-Tat/HIV-Luc (2 × 10^8^ PFU/mouse), an equal mixture of AdV.C3-Tat/HIV-Box A variants, or the individual AdV.C3-Tat/HIV-Box A variants (Gly or Ser) (2 × 10^8^ PFU/mouse). Data are from two separate experiments with 5 mice/treatment/experiment. (D) Mice were infected as in panel A. Mice were treated i.v. with AdV.C3-Tat/HIV-Luc (2 × 10^7^ PFU/mouse) or an equal mixture of AdV.C3-Tat/HIV-Box A variants (2 × 10^7^ PFU/mouse) on day 1, day 3, or day 5 post-infection. *N* = 5 mice/treatment group. (E) Mice were infected as in panel A. Mice were treated i.v. with AdV.C3-Tat/HIV-Luc (2 × 10^8^ PFU/mouse) or an equal mixture of AdV.C3-Tat/HIV-Box A variants (2 × 10^8^ PFU/mouse) at day 1, day 3, or day 5 p.i. *N* = 5 mice/treatment group.

Mice were infected with PR8 (LD_90_), then 24 h later, administered saline, 2 × 10^7^ PFU of AdV-C3-Tat/HIV-Luc (i.v.) or 2 × 10^7^ PFU of AdV.C3-Tat/HIV-Box A^Ser/Gly^ (an equal mix of Ser and Gly variants) by either i.v. or i.m. routes. We initially used a mixture of the AdV.C3-Tat/HIV-Box A^Ser^ and AdV.C3-Tat/HIV-Box A^Gly^ since we did not know if the two vectors would be equivalently protective, despite comparable expression. This dose is very low compared to doses used for vaccines or gene therapy (e.g., 10^9^ to 10^10^ PFU (64)). Neither saline- nor AdV-C3-Tat/HIV-Luc protected mice against the lethal PR8 challenge (Fig. 4A). By contrast, a single i.v. dose of the AdV.C3-Tat/HIV-Box A^Ser/Gly^ vectors significantly improved survival (to ~60%), while i.m. treatment AdV.C3-Tat/HIV-Box A only delayed death (blue line, Fig. 4A). When the i.m. dose of AdV.C3-Tat/HIV-Box A vectors was increased 10-fold to 2 × 10^8^ PFU/mouse, survival was enhanced to ~50%, approaching that induced by i.v. administration of 2 × 10^7^ PFU/mouse (compare Fig. S2 (2 × 10^8^ i.m.) with Fig. 4A).

These experiments were extended by comparing the responses of mice to influenza PR8 infection, followed on day 1 by i.v. treatment with 2 × 10^7^ PFU/mouse of the AdV.C3-Tat/HIV-Luc, AdV.C3-Tat/HIV-Box A^Ser/Gly^, AdV.C3-Tat/HIV-Box A^Ser^ only, or AdV.C3-Tat/HIV-Box A^Gly^ only. No mice survived treatment with control AdV.C3-Tat/HIV-Luc. Surprisingly, the mixture of the two variant vectors was more protective than the same dose of either variant vector alone (Fig. 4B). When the dose of the mixed and individual vectors was increased 10-fold and administered on day 1, the individual Box A Ser or Gly vectors protected mice comparably to the mixture (Fig. 4C). To assess whether treatment could be delayed and still protect, mice were infected then administered 2 × 10^7^ PFU/mouse of AdV.C3-Tat/HIV-Luc (day 1 only) or AdV.C3-Tat/HIV-Box A^Ser/Gly^ i.v. on days 1, 3, or 5 p.i. Figure 4D shows that AdV.C3-Tat/HIV-Box A^Ser/Gly^ treatment at day 1 p.i. elicited the same degree of protection seen in Fig. 4A and B. Delaying treatment until day 3 p.i. resulted in a significant increase in the time to death (*P* = 0.0179). No protection was observed if treatment was delayed until day 5 p.i. When the treatment dose was increased 10-fold, enhanced protection was observed when the vector mixture was administered on day 3, but not if treatment was delayed until day 5 (Fig. 4E). Thus, protection induced by AdV.C3-Tat/HIV-Box A^Ser^ and AdV.C3-Tat/HIV-Box A^Gly^ are dose- and time-dependent.

CR were similarly treated i.v. with AdV.C3-Tat/HIV-Box A^Ser^ and whole blood and organs were collected 1 day later for analysis of AdV DNA by qPCR. AdV DNA was detected primarily in blood, liver, spleen, and heart, with small intestine and lung as secondary sites (Table S1). Thus, the AdV vector disseminates throughout the body upon i.v. delivery. Together, the data show that our non-replicating AdV.C3-Tat/HIV-Box A vectors are inflammation-regulated and sufficiently active *in vitro* and *in vivo* to induce rBox A in response to potent non-infectious or infectious inflammatory stimuli (e.g., LPS or influenza) and AdV.C3-Tat/HIV-Box A constructs protect against influenza-induced disease.

### Effect of AdV.C3-Tat/HIV-Box A therapy on the inflammatory response to influenza

**Fig 5:**
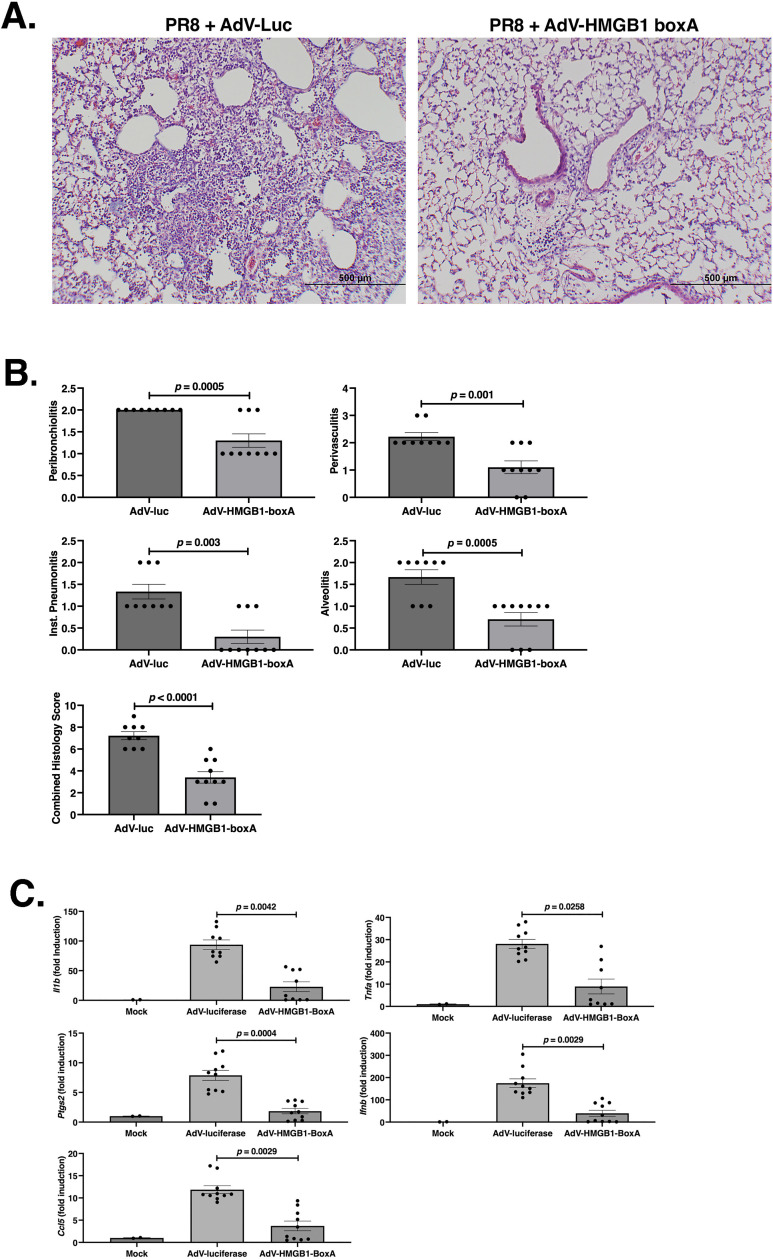
C57BL/6J mice were infected on day 0 with PR8 (LD_90_). Twenty-four hours later, mice were treated i.v. with saline (mock), AdV.C3-Tat/HIV-Luc, or an equal mixture of AdV.C3-Tat/HIV-Box A variants (2 × 10^7^ PFU). (A) Representative pathology of lung sections derived from mice treated therapeutically with AdV.C3-Tat/HIV-Luc or AdV.C3-Tat/HIV-Box A 24 h after infection with PR8 (LD_90_) at 5 days p.i. (B) Quantification of lung histopathology scores for the individual mice including a combined histology score. (C) Quantification of lung cytokine mRNA levels by qRT-PCR for the individual mice. (B and C) Each symbol represents one mouse.

Mice were infected and treated i.v. as in Fig. 4A. Five days p.i., a time just before control mice begin to die, lungs were harvested and pathology was blindly scored in H&E-stained sections as detailed in Materials and Methods (65). Figure 5A shows representative photomicrographs of lung sections. Mice treated therapeutically with AdV.C3-Tat/HIV-Luc (left) exhibited much greater lung inflammation than mice treated with AdV.C3-Tat/HIV-Box A^Ser/Gly^ (right). Mice treated with 2 × 10^7^ PFU/mouse of AdV.C3-Tat/HIV-Box A^Ser/Gly^ exhibited significantly reduced pathology for each parameter as well as for the combined pathology score (Fig. 5B).

The effect of AdV.C3-Tat/HIV-Box A therapy on the cytokine response induced by influenza was also measured. Mice that received saline only (mock) had low levels of lung cytokine mRNA, in contrast to PR8-infected mice treated with AdV.C3-Tat/HIV-Luc (Fig. 5C). PR8-infected mice treated with AdV.C3-Tat/HIV-Box A^Ser/Gly^ had significantly decreased lung inflammatory cytokine mRNA observed compared to AdV.C3-Tat/HIV-Luc treatment. At this same time, levels of AdV vectors were comparable (Fig. S3).

**Fig 6:**
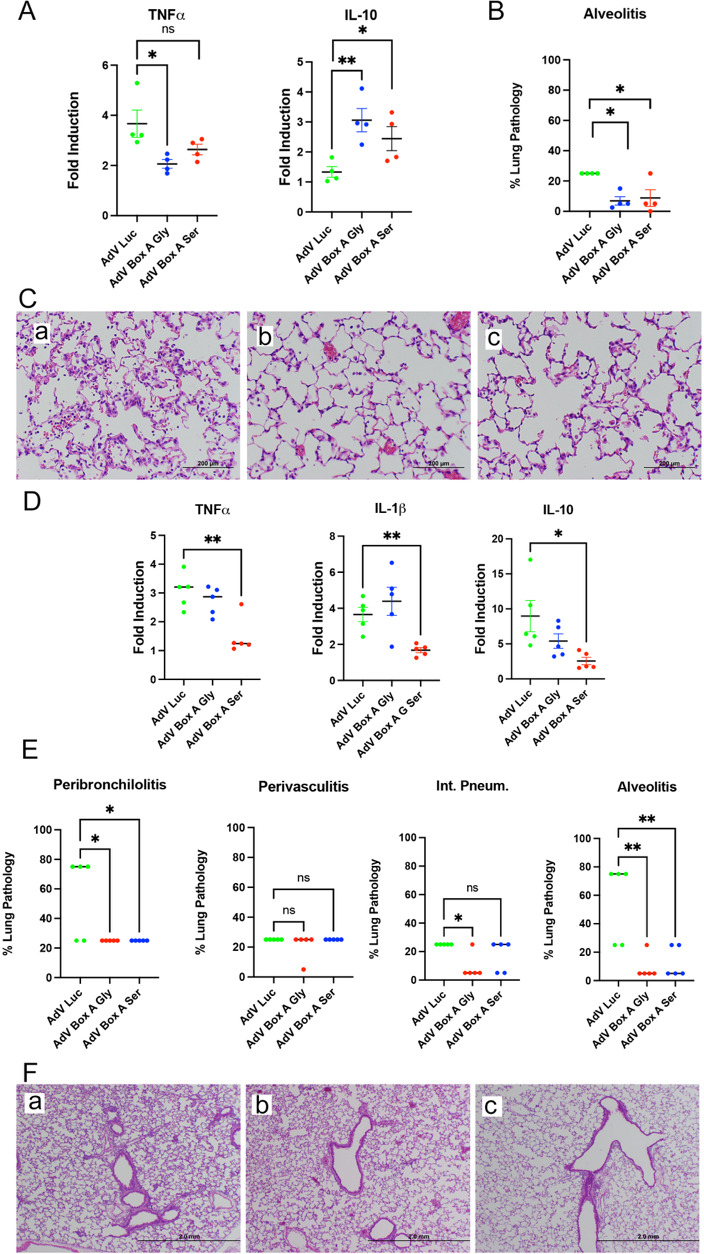
CR were infected i.n. on day 0 with A(H3N2) (10^7^ TCID_50_/CR). On day 1 p.i., CR were treated by i.v. injection with 10^7^ PFU/CR of AdV.C3-Tat/HIV-Luc, AdV.C3-Tat/HIV-Box A^Gly^ or AdV.C3-Tat/HIV-Box A^Ser^. CR were then sacrificed on 3 and 6 days p.i. to analyze lung cytokine mRNA expression and lung histopathology. (A) mRNA expression of TNFα and IL-10 in lung samples of CR sacrificed on day 3 p.i. (*n* = 4/treatment). (B) Score of alveolitis at 3 days p.i. for the different groups of CR (*n* = 4/treatment). (C) Representative microscopic images of lungs of CR treated with AdV.C3-Tat/HIV-Luc (a), AdV.C3-Tat/HIV-Box A^Gly^ (b), or AdV.C3-Tat/HIV-Box A^Ser^ (c) showing reduced numbers of cells in the alveolar spaces of CR treated with AdV.C3-Tat/HIV-Box A vectors. Magnification, 200×. (**D**) mRNA expression of TNFα, IL-1β, and IL-10 in lungs of CR harvested at day 6 p.i. (*n* = 5/treatment) (**E**) Pathology scores for lungs of individual CR for peribronchioliis, perivasculitis, interstitial pneumonia, and alveolitis (*n* = 5/treatment). (F) Representative microscopic images of lungs of CR treated with AdV.C3-Tat/HIV-Luc (a), AdV.C3-Tat/HIV-Box A^Gly^ (b), or AdV.C3-Tat/HIV-Box A^Ser^ (c) showing reduced peribronchiolitis in animals treated with AdV.C3-Tat/HIV-Box A vectors. Magnification, 40×. *n* = 4–5/group; Student *t* test, **P < 0.01; **P < 0.05.

CR were infected i.n. with influenza A(H3N2) (10^7^ TCID_50_/CR) on day 0. On day 1 p.i., groups of CR were treated with AdV.C3-Tat/HIV-Luc, AdV.C3-Tat/HIV-Box A^Gly^, or AdV.C3-Tat/HIV-Box A^Ser^ (10^7^ PFU/animal, i.v.), and sacrificed on either day 3 or day 6 p.i. (Fig. 6). Figure 6A shows that just 2 days post-treatment, levels of TNFα mRNA were significantly reduced in CR that received AdV.C3-Tat/HIV-Box A^Gly^ vector, with a similar but non-significant trend for those treated with AdV.C3-Tat/HIV-Box A^Ser^. Conversely, levels of anti-inflammatory IL-10 mRNA were upregulated by treatment with AdV.C3-Tat/HIV-Box A^Gly^ or AdV.C3-Tat/HIV-Box A^Ser^. No significant change in the production of influenza M protein mRNA was detected (data not shown), indicating that influenza replication was not affected at this time point. Lung histopathology (Fig. 6B) at day 3 p.i. revealed significantly reduced alveolitis in CR treated with either AdV.C3-Tat/HIV-Box A^Gly^ and AdV.C3-Tat/HIV-Box A^Ser^ (Fig. 6B and C).

In CR lungs harvested at day 6 p.i., levels of TNFα, IL-1β, and IL-10 mRNA were significantly reduced only when treated with AdV.C3-Tat/HIV-Box A^Ser^ (Fig. 6D). Peribronchiolitis and alveolitis scores were significantly decreased in CR treated with either Box A variant, whereas interstitial pneumonia was significantly reduced only in CR that had been treated with AdV.C3-Tat/HIV-Box A^Gly^. By day 6, perivasculitis was not resolved in any group (Fig. 6E). Figure 6F illustrates representative images from CR treated with AdV.C3-Tat/HIV-Luc (i), AdV.C3-Tat/HIV-Box A^Gly^ (ii), or AdV.C3-Tat/HIV-Box A^Ser^ (iii) showing reduced peribronchiolitis in CR treated with both Box A variants. Thus, both AdV.C3-Tat/HIV-Box A^Gly^ and AdV.C3-Tat/HIV-Box A^Ser^ exert anti-inflammatory effects in response to human influenza A(H3N2) infection of CR.

## DISCUSSION

Increased circulating HMGB1 correlates with many inflammatory diseases, that is, sepsis, viral respiratory infections, traumatic brain injury, systemic lupus erythematosus, Alzheimer’s disease, cancer, and others (42–46). HMGB1 has three cysteine residues, but only the disulfide (cys23 and cys45) isoform is a TLR4 DAMP, binding MD-2 at a site distinct from LPS. Blocking the HMGB1 binding to MD-2 with P5779 blocked HMGB1-mediated, but not LPS-induced TLR4 signaling (32). In addition, monoclonal antibody 2G7 (directed against Box A), rBox A, and glycyrrhizin (a molecule that binds HMGB1) antagonize HMGB1-mediated signaling in models of inflammation (66–69), yet have not advanced beyond pre-clinical studies. We hypothesized that an inflammation-inducible viral vector might facilitate more sustained expression of therapeutic transgenes that dissipate as inflammation wanes (70).

**Fig 7:**
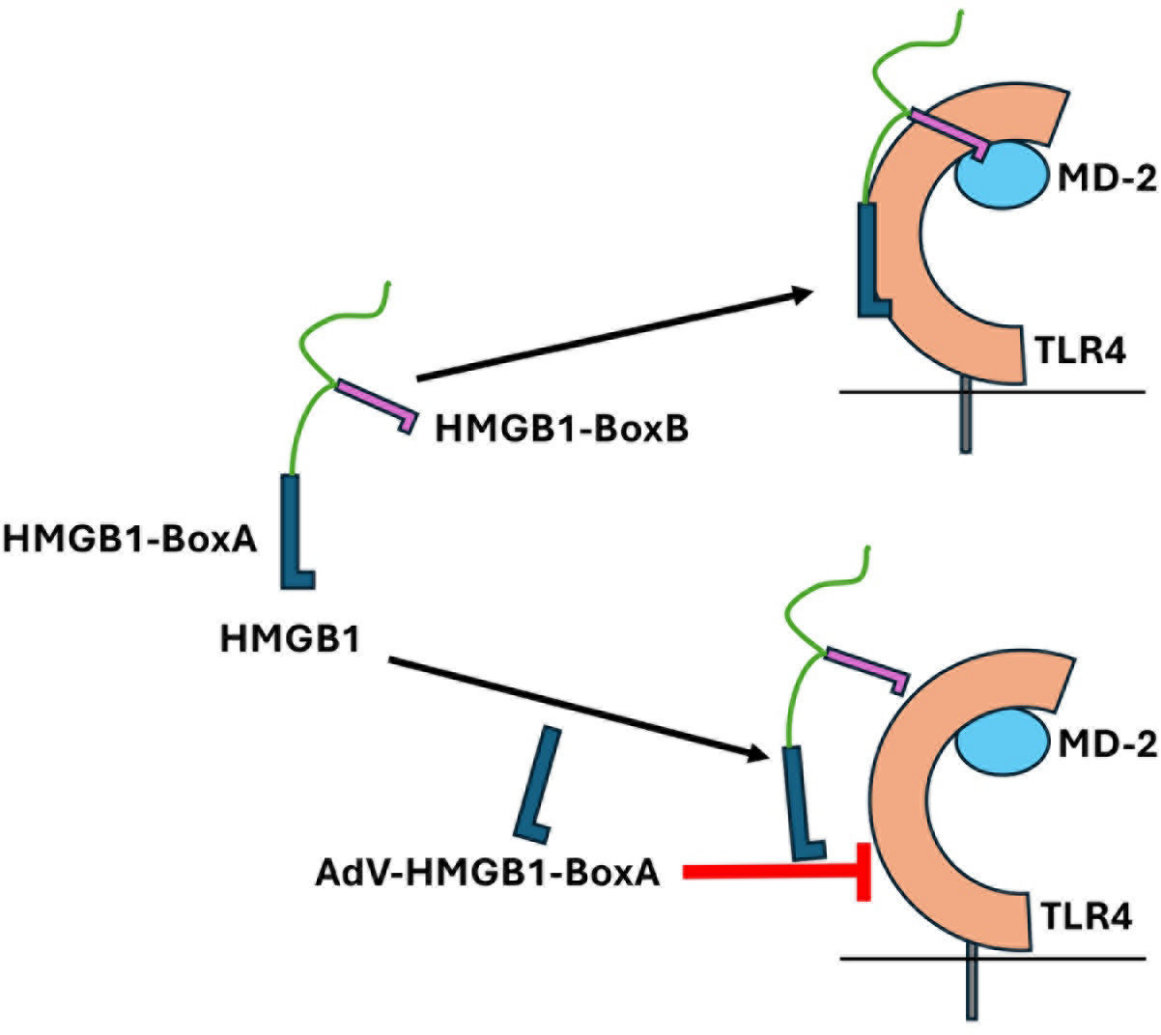
Model. Intact HMGB1 contains two domains, HMGB1 Box A and HMGB1 Box B. The HMGB1 Box A domain binds to TLR4, while the HMGB1 Box B domain binds MD-2 to elicit TLR4 signaling (top image). In response to an inflammatory stimulus, the Adv.C3-Tat/HIV-Box A vector produces rBox A that competitively inhibits the binding of the intact protein HMGB1’s Box A domain to TLR4 and precludes its Box B from binding to MD-2 (bottom image).This results in disruption of TLR4-mediated signaling (modified from reference 34).

He et al. (34) showed that rBox A competitively antagonizes the binding of intact HMGB1 to TLR4, causing its release from both TLR4 and MD-2, thereby significantly reducing TLR4-mediated signaling. Our data support this model (Fig. 7) and indicate that sufficient HMGB1 Box A is synthesized from our AdV vectors in an inflammation-inducible fashion to blunt the effect of HMGB1 in rodent models of LPS and influenza challenge.

In experimental endotoxicity and sepsis, where bacterial-derived rBox A or P57799 was used to antagonize HMGB1-mediated signaling, high concentrations, and multiple doses were required (17, 30). We sought proof of principle that therapy with an AdV vector that elicits inflammation-inducible rBox A would mitigate ALI in two well-established rodent models of influenza. We took advantage of a non-replicating AdV vector to respond to inflammation to drive the production of specific proteins (55–59). Specifically, luciferase was expressed in AdV-treated mice challenged with LPS or turpentine (55, 56), and when modified to express IL-10 or IL-1RA, and administered into the joints of arthritic rodents, lessened inflammation (58, 59). When we substituted the HMGB1 Box A sequence for sequences encoding luciferase, IL-10, or IL-1RA, Box A-His protein was secreted in response to inflammatory stimuli *in vitro, ex vivo*, or *in vivo*. AdV.C3-Tat/HIV-Box A^Ser^ failed to induce significant inflammation in CR at high doses; however, when administered therapeutically after influenza infection, lethality, and ALI were mitigated in mice, as was ALI in CR, accompanied by a significant reduction in proinflammatory cytokine responses. Intravenous was more effective than i.m. treatment, but increasing the dose i.m. compensated to improve survival and may be related to different kinetics of rBox A expression or cellular tropism of the AdV vector. Unexpectedly, an equal mixture of the AdV.C3-Tat/HIV-Box A^Ser^ and AdV.C3-Tat/HIV-Box A^Gly^ variants, presumably producing both glycosylated and non-glycosylated forms of rBox A, provided greater protection at a lower dose (2 × 10^7^ PFU/mouse) than an equivalent dose of either variant alone. Nonetheless, increasing the dose of either variant alone 10-fold improved survival equivalent to that of the mixed vectors. While we do not know the mechanism underlying this observation, it is possible that differentially glycosylated rBox A proteins are released in different compartments *in vivo*, antagonize topologically different TLR4/MD-2 receptors, or that the two different rBox A variants interact synergistically to compete with HMGB1 for binding to TLR4. Since rBox A blocks RAGE-dependent endocytosis (71), our approach may also apply to RAGE-mediated diseases, in contrast to P5779 which is specific for TLR4/MD-2 (32). Together, treatment of influenza-infected mice/CR with the inflammation-inducible AdV.C3-Tat/HIV-Box A decreased lethality, pathology, and proinflammatory cytokine expression.

The protection from lethal PR8 challenge by TLR4 or HMGB1 antagonists exceeded that reported herein with the AdV.C3-Tat/HIV-Box A vector (95%–100% vs. ~60%). This may be attributable to the fact that the former was administered at high doses daily for each of 5 days, in contrast to a single injection of AdV.C3-TAT/HIV-Box A. This was done intentionally to minimize the host response to the vector itself. Studies are ongoing to further optimize vector delivery, timing, and route of administration.

This approach is early in development but has significant potential for treating multiple HMGB1-mediated diseases. To demonstrate proof-of-concept, we used a human adenovirus type-5 vector (HAdV-C5) due to the ease of genetic engineering, the ability to grow it to high titers, and well-established dosing regimens for rodents. However, humans may have pre-existing immunity to this AdV serotype, which could negate the therapeutic effect (72). In addition, i.v administration of AdV5 in humans warrants caution due to off-target interactions with coagulation factors and other blood components, possibly resulting in dose-limiting toxicity (73). Despite this, this inducible therapeutic transgene cassette is compatible with many other vector systems such as rare serotype AdVs (>200 vectors exist with low seroprevalence in humans (72, 74)), adeno-associated vectors, or lentiviruses (75). Such platforms could be engineered and tailored to specific inflammatory disease conditions in the future, including those focused on localized delivery rather than systemic delivery.

## MATERIALS AND METHODS

### Construction of AdV.C3-Tat/HIV-Box A and AdV.C3-Tat/HIV-Luc

Adenovirus C3-tat/HIV-luc (provided by Dr. Robert Munford (56)) was transduced into HEK-293A cells that were grown in DMEM with 10% fetal bovine serum, 1% amphotericin B, 1% penicillin/streptomycin, and 1% glutamax (ThermoFisher, 35050061). Transductions were done using 50 µL of virus stock in opti-MEM (ThermoFisher, 31985-070) for 1 h in a 75% confluent T225 culture flask at 37°C. After 2 days, viruses were harvested by collecting the medium and lysing cells using three freeze-thaw cycles in PBS. Lysates were centrifuged (10,000 rpm, 15′ at 4°C). Cleared lysates’ supernatant and medium were pooled, aliquoted, and stored at −70°C as AdV.C3-Tat/HIV-Luc viral stocks.

### Amplification of the AdV transgene

DNA was extracted from virus stocks using a QIAamp DNA Mini Kit (Qiagen, 51304). DNA was used as a template for PCR amplification of the two-component transgene (C3-Tat/HIV(LTR)-Luc), with the following primers that incorporate KpnI and XhoI sites: FWD, 5′-CAGCTTTAAAGGTACCCGGGGATCCAGACATGATAAGATAC-3′; REV, 5′-TAGCTGATATCCTCGAGATCGATGATACCCAATTCAACAGGC-3′.

Reactions were carried out using GoTaq long PCR (Promega, M4021). Amplified fragments (cassette) were agarose gel purified and extracted (New England Biolabs, Monarch 1020S).

### Cloning of C3-Tat/HIV(LTR)-Luc cassette into Gateway pENTR 1A dual selection vector (Invitrogen, A10462)

Box A expression cassettes were cloned into pENTR 1A dual selection vector (ThermoFisher, A10462). Plasmids were purified from the T1 bacteria (Invitrogen, A10460) using a plasmid maxi-prep kit (Qiagen, 12362). Amplified PCR products were cloned into pENTR 1A using KpnI and XhoI restriction sites.

Primers for sequencing (5′→3′) were as follows: CACCACTGCTCCCATTCATCAGTTCC, GATCGCCGTGTAATTCTAGAGGATC, CCTTACTTCTGTGGTGTGACATAATTGG, CCTTTCTTTATGTTTTTGGCGTCTTCC, and GTAACATCAGAGATTTTGAGACA.

### Transfer of the HMGB1-Box A (Ser or Gly) transgene into the cassette

pcDNA3.1 plasmids containing the engineered “IgK leader-Box A-6×His” constructs were synthesized using synthesis services (GeneWhiz) and amplified using primers that included a NcoI or NotI site matching cloning sites in the pENTR 1A.C3-Tat/HIV(LTR)-Luc. Sequencing confirmed the cloning of Box A inserts (accession number X12597.1, HMGB1 sequence human mRNA and nucleotides 53–319 for Box A), resulting in three pENTR 1A with the two-component inducible-expression system: C3-Tat/HIV-Luc, C3-Tat/HIV-Box A^Ser^, and C3-Tat/HIV-Box A^Gly^.

### Propagation of AdV.C3-Tat/HIV-Box A and AdV.C3-Tat/HIV-Luc constructs

Recombinant AdV5 genomes (E1/E3 deleted) were produced by *in vitro* homologous recombination using LR clonase and gateway technology (Life Technologies). Plasmids containing genetically modified adenoviral genomes were fully sequence confirmed using PlasmidSaurus Inc., followed by P*ac*I digestion to release the viral genome, and transfection into T-Rex-293 cells (Life Technologies). Viruses were scaled up, released from cells by freeze-thaw cycles, purified using two rounds of CsCl ultracentrifugation, and titrated (76). Physical viral particle titers were determined using a microBCA assay (63, 77). Expression of rBox A^Ser^ and rBox A^Gly^ was confirmed in transient transfection experiments using Expi293F cells (ThermoFisher).

### Mice and CR

Six-week-old WT C57BL/6J mice were purchased from Jackson Laboratory (Bar Harbor, ME). Six- to 8-week-old RAGE^−/−^ mice (provided by Dr. Ann Marie Schmidt, NYU) were bred in-house at UMB. Four- to 6-week-old female and male CR (~100 g), seronegative for adventitious respiratory viruses, were obtained from SBI’s inbred colony.

For *in vitro* studies, thioglycollate-elicited peritoneal CR Mϕ was cultured as reported (78). BAL Mϕ were collected by washing the entire lung block with 3 mL cold saline, three times (78).

### Influenza viruses

Mouse-adapted A(H1N1) influenza A/PR/8/34 virus (“PR8”) (ATCC, Manassas, VA) was kindly provided by Dr. Donna Farber (Columbia University). The human A(H3N2) virus was provided by the Centers for Disease Control and Prevention and propagated in Madin-Darby canine kidney (MDCK) cells.

### Mouse and CR virus challenge and treatment

For survival experiments, mice were infected with an LD_90_ of PR8 (~7500 TCID_50_ i.n., 25 µL/nares) (14, 17). One day after infection, mice received saline, AdV.C3-Tat/HIV-Luc (2 × 10^7^ PFU), an equal mixture of AdV.C3-Tat/HIV-Box A variants, i.m or i.v., or the individual AdV.C3-Tat/HIV-Box A variants (Gly or Ser) (2 × 10^7^ PFU or 2 × 10^8^ PFU) by i.v. injection. For time course studies, mice were infected on day 0 with PR8 (LD_90_), then treated with AdV.C3-Tat/HIV-Luc or an equal mixture of AdV.C3-Tat/HIV-Box A variants (2 × 10^7^ PFU or 2 × 10^8^ PFU) i.v. on days 1, 3, or 5 p.i. For tissue analysis, mice were infected on Day 0 with PR8 (LD_90_). Day 1 p.i., mice were treated with saline (mock), AdV.C3-Tat/HIV-Luc, or an equal mixture of AdV.C3-Tat/HIV-Box A variants (2 × 10^7^ PFU) i.v. On Day 5 p.i., lungs were harvested for histology and gene expression. WT and RAGE^−/−^ mice were infected with PR8 (LD_90_). Mice were monitored daily for 14 days.

CR were infected i.n. with 100 µL (10^7^ TCID_50_) of A(H3N2) virus. Treatments were performed retro-orbitally under isoflurane anesthesia. In some experiments, AdV vectors were administered i.n. Animals were euthanized by CO_2_ asphyxiation.

### Western blot

For secreted protein, culture supernatants were collected with protease inhibitor (CST, 5871S), and incubated with Ni-Sepharose FF-6 beads (Cytiva, 17531806, 50% slurry) for 2 h at RT. Washed beads were incubated with sample buffer and 10× reducing agent (Invitrogen, NP0007, NP0009), boiled, and loaded onto a 4%–12% Bis-Tris gel (NuPAGE, Invitrogen). Cell lysates were collected in RIPA buffer with protease inhibitor and centrifuged at 14,000 rpm for 10 min. Supernatants were mixed with 10× reducing agents and loaded onto gels (NuPAGE, Invitrogen). Ponceau stain (Sigma-Aldrich, P7170) reflected protein loading. MW markers (Cytiva, RPN800E) and rBox A produced in Expi293F cells (ThermoFisher, A14635) transfected with a pcDNA3.1 containing the Box A sequence were used to confirm MW and antibody specificity. Primary anti-6x His (ThermoFisher, MA1-21315) or anti-HMGB1 (Abnova, H00003146-M08) antibodies (1:1,000 dilution) and a sheep anti-mouse IgG HRP conjugate (Cytiva NA931V) were used for detection. Blots were developed using ECL substrate and film (Cytiva, RPN3004, 28906838).

### Luciferase assays

Mϕ were lysed in Glo-lysis buffer (Promega, E266A), mixed with equal volumes of Bright-Glo reagent (Promega, E2610), and assayed on a luminometer (Promega, GM2000 Glomax Navigator).

For tissue luciferase assays, ~200 mg tissue was placed into 1.5 mL of Glo-lysis buffer with two steel beads, homogenized at 50 Hz on a Qiagen tissuelyser LT, centrifuged, and 100 µL of supernatant mixed with 100 µL of Bright-Glo reagent to assay.

### Tissue distribution of AdV.C3-Tat/HIV-Box A following i.v. injection of CR

CR were treated retro-orbitally with 1 × 10^7^ PFU of AdV.C3-Tat/HIV-Box A^Ser^. One day post-treatment, CR were bled, euthanized, and organs dissected for the detection of the AdV genome using qPCR with two different specific primer sets, the +AdV set (79) and the +Hex set (80). Tissues of uninfected animals showed Ct values of 40.

### Histology and staining

Lungs were inflated and fixed with 4% PFA. Lung sections (5 µm) were stained with H&E. Four parameters were scored independently from 0 to 4 for each section: peribronchiolitis (primarily lymphocytes, surrounding a bronchiole), perivasculitis (primarily lymphocytes, surrounding a blood vessel), alveolitis (within alveolar spaces), and interstitial pneumonia (increased thickness of alveolar walls). Slides were randomized and blindly scored. Data are shown as individual scores for each parameter or a cumulation of the four parameters (65).

### Quantitative real-time PCR

Total murine RNA isolation and quantitative real-time (qRT)-PCR were performed as previously described (14, 81, 82). Levels of mRNA for specific mouse genes were normalized to the level of the housekeeping gene, *Hprt*, in the same samples (83). For CR qRT-PCR (41, 84), amplifications were performed on a Bio-Rad iCycler (MyiQ Single Color). Each gene was normalized to β-actin mRNA as a housekeeping gene (83). Data are expressed as “fold induction” (2^−ΔΔCt^) over mock-treated animals.

### Statistics

Statistical differences between the two groups were determined by unpaired, two-tailed Student’s *t* test with significance set at *P* < 0.05. For >3 groups, analysis was done by one-way ANOVA followed by a Tukey’s post hoc test with significance determined at *P* < 0.05. For survival studies, a log-rank (Mantel-Cox) test was used.

## Data Availability

All primary data from which the figures in this paper and the supplemental materials are deposited in the OSF public database at https://osf.io/dgyxw/. All novel materials described will be provided through a licensing agreement or a materials transfer agreement with the University of Maryland, Baltimore.

## SUPPLEMENTAL MATERIAL

Supplemental tables and figures mbio.03387-24-s0001.pdf Table S1 and Fig. S1 to S3.

ASM does not own the copyrights to Supplemental Material that may be linked to, or accessed through, an article. The authors have granted ASM a non-exclusive, world-wide license to publish the Supplemental Material files. Please contact the corresponding author directly for reuse.
