# Supplementary material for: An adenoviral vector encoding an inflammation-inducible antagonist, HMGB1 Box A, as a novel therapeutic approach to inflammatory diseases
Source: mBio. 2024 Dec 19;16(2):e03387-24. doi: 10.1128/mbio.03387-24 (PMC11796352; doi:10.1128/mbio.03387-24)
Supplement: Supplemental tables and figures — Table S1 and Fig. S1 to S3. [file mbio.03387-24-s0001.pdf]

## SUPPLEMENTARY MATERIALS

| Tissue | Samples (n) | (+) AdV Primer | (+) Hexa Primer |
|---|---|---|---|
| Blood | 2 | 2/2 | 2/2 |
| Liver | 3 | 3/3 | 3/3 |
| Spleen | 3 | 3/3 | 3/3 |
| Kidney | 3 | 0/3 | 1/3 |
| Small Int. (proximal) | 3 | 1/3 | 2/3 |
| Small Int. (medium) | 3 | 0/3 | 0/3 |
| Small Int. (terminal) | 3 | 0/3 | 0/3 |
| Large Int. | 3 | 0/3 | 0/3 |
| Heart | 3 | 3/3 | 3/3 |
| Lung | 3 | 1/3 | 3/3 |
| Brain | 3 | 2/3 | 2/3 |
| Thymus | 3 | 0/3 | 1/3 |
| Gonads | 3 | 0/3 | 0/3 |
| Vessel | 3 | 0/3 | 0/3 |
| Peritoneum | 3 | 1/3 | 0/3 |
| Tail | 3 | 0/3 | 0/3 |
| Bone Marrow | 3 | 0/3 | 2/3 |
| Mesenterium | 3 | 0/3 | 0/3 |

**Table SI.** Distribution of AdV.C3-Tat/HIV-Box A in CR. CR were treated i.v. with $10^7$ PFU/CR of AdV.C3-Tat/HIV-Box $A^{Ser}$. One day after treatment, CR were bled, sacrificed, and dissected to obtain samples of the indicated tissues. Total genomic DNA was purified from each of the indicated organs, subjected to qPCR for the detection of AdV genome using two different sets of primers . Organs in red indicate those with consistent detection of AdV DNA. Number of samples tested from each indicated organ. Some samples are not equivalent due to sampling variation in different locations within the organ.

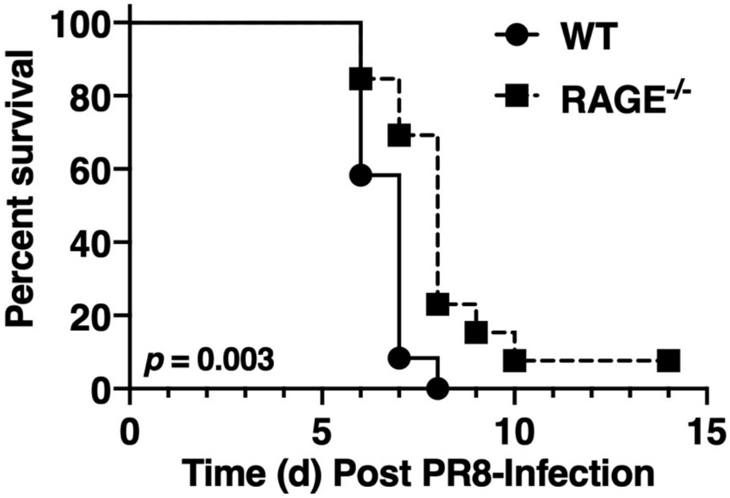


**Figure S1**. Extended meantime to death of RAGE$^{-/-}$ mice to PR8 infection. C57BL/6J and RAGE$^{-/-}$ mice were infected with influenza PR8 ($LD_{90}$) i.n. and monitored daily for survival for 14 days post-PR8 infection. Data represent the combined results of 2 separate experiments (WT, n=12; RAGE$^{-/-}$, n=13). Data analyzed by log rank Mantel-Cox test.

.

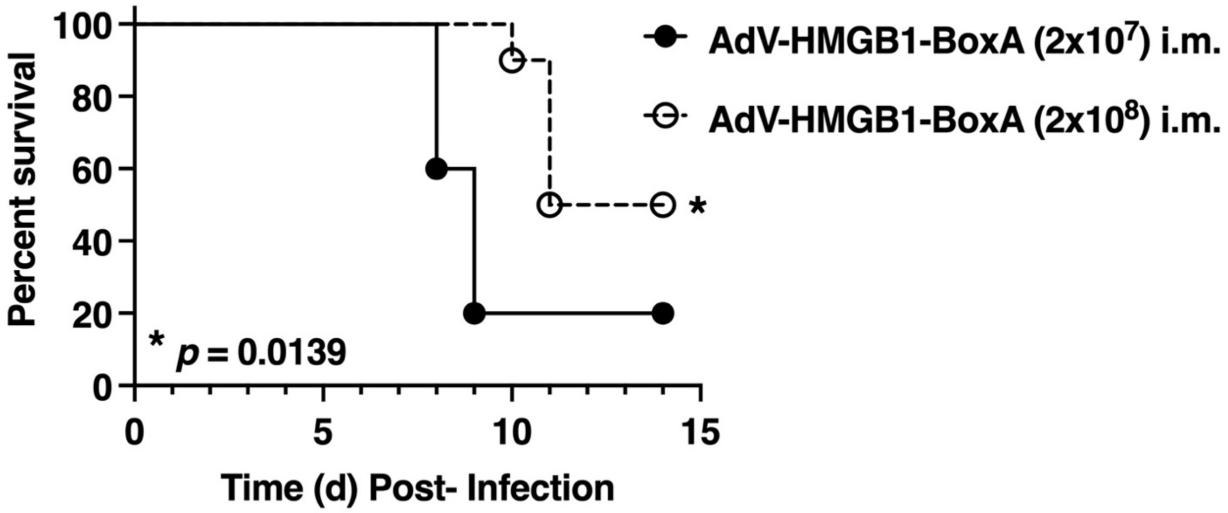


**Figure S2.** C57BL/6J mice were infected on Day 0 with PR8 ($LD_{90}$). Twenty-four hours later, mice were treated i.m. with an equal mixture of AdV.C3-Tat/HIV-Box A variants at either $2X10^7$ PFU/mouse or $2X10^8$ PFU/mouse. Mice were monitored for survival for 14 days. C57BL/6J mice were infected on Day 0 with PR8 ($LD_{90}$). Data are from 2 separate experiments with 4-6 mice/treatment/experiment. Data analyzed by log rank Mantel-Cox test.

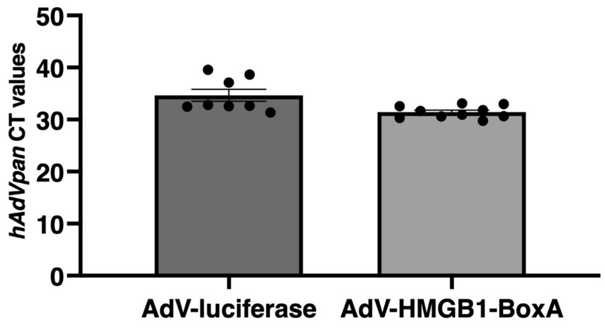


**Figure S3.** The lung samples analyzed in Figure 5C for cytokine mRNA expression were also analyzed for detection of AdV DNA.
